# Hitchhiking motility of *Staphylococcus aureus* involves the interaction between its wall teichoic acids and lipopolysaccharide of *Pseudomonas aeruginosa*

**DOI:** 10.3389/fmicb.2022.1068251

**Published:** 2023-01-05

**Authors:** Chao-Chin Liu, Mei-Hui Lin

**Affiliations:** ^1^Graduate Institute of Biomedical Sciences, Chang Gung University, Taoyuan, Taiwan; ^2^Department of Medical Biotechnology and Laboratory Science, College of Medicine, Chang Gung University, Taoyuan, Taiwan; ^3^Department of Laboratory Medicine, Chang Gung Memorial Hospital, Taoyuan, Taiwan

**Keywords:** *Staphylococcus aureus*, *Pseudomonas aeruginosa*, TagO, teichoic acids, lipopolysaccharide, hitchhiking motility

## Abstract

*Staphylococcus aureus*, which lacks pili and flagella, is nonmotile. However, it hitchhikes motile bacteria, such as *Pseudomonas aeruginosa*, to migrate in the environment. This study demonstrated that the hitchhiking motility of *S. aureus* SA113 was reduced after the *tagO*, which encodes an enzyme for wall teichoic acids (WTA) synthesis, was deleted. The hitchhiking motility was restored after the mutation was complemented by transforming a plasmid expressing TagO into the mutant. We also showed that adding purified lipopolysaccharide (LPS) to a culture that contains *S. aureus* SA113 and *P. aeruginosa* PAO1, reduced the movement of *S. aureus*, showing that WTA and LPS are involved in the hitchhiking motility of *S. aureus*. This study also found that *P. aeruginosa* promoted the movement of *S. aureus* in the digestive tract of *Caenorhabditis elegans* and in mice. In conclusion, this study reveals how *S. aureus* hitchhikes *P. aeruginosa* for translocation in an ecosystem. The results from this study improve our understanding on how a nonmotile pathogen moves in the environment and spreads in animals.

## Introduction

Microorganisms in an ecosystem often compete for resources to improve their survival (Ghoul and Mitri, 2016). One of the well-known examples occurs in a cystic fibrosis infection model where *Pseudomonas aeruginosa* changes the physiology and inhibits the growth of *Staphylococcus aureus* (Mashburn et al., 2005; Pernet et al., 2014); nevertheless*, S. aureus* prevents growth inhibition by *P. aeruginosa* through changes in metabolic pathways and growth characteristics (Hoffman et al., 2006; Biswas et al., 2009; Briaud et al., 2019). Another example regarding interspecies interaction is that *Candida albicans* enhances biofilm formation, antibiotic resistance and virulence of *S. aureus* through activating the staphylococcal *agr* quorum sensing system (Todd et al., 2019). Additionally, the expression of *C. albicans* drug resistant gene is upregulated during co-culture with *S. aureus* (Hu et al., 2021).

Motility is crucial for bacteria to escape dangers and search for nutrients in the environment, which are important to their virulence and pathogenicity (Matilla and Krell, 2018). Recent studies have demonstrated that nonmotile bacteria are capable of attaching motile bacteria to move and relocate to favorable niches (Hagai et al., 2014; Samad et al., 2017). For instance, *Xanthomonas perforans*, a weak swarmer, produces substances allowing the organism to attach to a motile bacterium, *Paenibacillus vortex*, to swarm efficiently on the surface of host plants (Hagai et al., 2014). Another example is that swarming *P. vortex* carries antibiotic-resistant *Escherichia coli* to help the organism against antibiotic stress (Finkelshtein et al., 2015). These studies indicate that microbes in a community often using hitchhiking strategy to migrate and overcome stresses in the environment.

*Staphylococcus aureus* is nonmotile due to the lack of flagella and pili (Pollitt and Diggle, 2017). However, this organism does move on soft agar surface *via* spreading (Kaito and Sekimizu, 2007; Lin et al., 2016). Its movement is mediated through the accumulation of water in a colony and the production of biosurfactants termed phenol soluble modulins (PSMs; Kaito and Sekimizu, 2007; Tsompanidou et al., 2013; Lin et al., 2016), which weakens the surface tension of the water, allowing water to flood on the agar surface and spreading of the organism (Lin et al., 2016). Meanwhile, in an ecosystem, *S. aureus* has opportunities to interact with neighboring microbes. *Staphylococcus aureus* and *P. aeruginosa* are two human pathogens often sharing the same niche (Pastar et al., 2013; DeLeon et al., 2014). A recent study demonstrated that swimming *P. aeruginosa* promotes dispersion of *S. aureus* (Samad et al., 2017). However, the mechanism underlying the interaction between *S. aureus* and *P. aeruginosa* remains unclear. This study demonstrated that *S. aureus* hitchhikes *P. aeruginosa via* interaction between wall teichoic acids (WTA) of *S. aureus* and lipopolysaccharide (LPS) of *P. aeruginosa*. The hitchhiking motility of *S. aureus* prompted by *P. aeruginosa was* also observed *in vivo* in a *Caenorhabditis elegans* model and a mouse model.

## Materials and methods

### Bacterial strains, plasmids, and culture conditions

*Staphylococcus aureus* SA113 (ATCC35556) was used for the study of hitchhiking motility (Herbert et al., 2010; Tsompanidou et al., 2011). A Δ*tagO* mutant of *S. aureus* SA113 contains a deletion in *tagO* and does not produce WTA (Weidenmaier et al., 2004). For complementing the Δ*tagO* mutation, the strain was transformed with a TagO-expressing plasmid, pHY-tagO. *Pseudomonas aeruginosa* PAO1 and its isogenic mutant Δ*fliA*, a flagellum-defective mutant (Lo et al., 2016) were kindly provided by Professor Hwan-You Chang. *Escherichia coli* EPI300 (Epicentre Technologies, Wisconsin, United States) and *S. aureus* RN4220, a restriction-deficient strain (Nair et al., 2011), were used as hosts for cloning. *Staphylococcus aureus* strains were transformed with pCtuf-ppmch (Mauthe et al., 2012), which encodes mCherry and exhibits red fluorescence. *Staphylococcus aureus* CGL1190, a bioluminescent strain that constitutively expresses a luciferase reporter. Plasmid pHY-tagO was constructed by inserting a PCR-amplified DNA fragment containing the *tagO* from *S. aureus* SA113. The fragment was amplified with primers 5′-CGGTCTAGATAGCACTTGTTACTGCAGCA and 5′-TTACCCGGGATCCCATACAGCTATGCTTT and digested with XbaI and SmaI and finally inserted into the same sites in pHY300pLK (TaKaRa Bio) to generate pHY-tagO. Bacteria were cultured in tryptic soy broth (TSB) and tryptic soy agar (TSA; Oxoid). Mannitol salt agar (BD Difco) was used for differentiating and enumerating *S. aureus* colonies. Antibiotic-resistant colonies were selected on media that contained ampicillin (100 μg/ml), chloramphenicol (10 μg/ml), kanamycin (50 μg/ml), and tetracycline (5 μg/ml).

### Construction of *Staphylococcus aureus* CGL1190

To generate a bioluminescent *S. aureus* strain, a temperature-sensitive plasmid pRP1190 (Plaut et al., 2013), which contains a modified *luxBADCE* operon from *Photorhabdus luminescens*, was transformed into *S. aureus* SA113. Integration of plasmids into bacterial chromosomes by homologous recombination was performed according to a method described previously (Plaut et al., 2013). The plates were imaged using an *in vivo* Imaging System (IVIS Lumina III, PerkinElmer, Waltham, Massachusetts, United States). Luminescent colonies were selected, and the integration site was confirmed by PCR.

### Assaying hitchhiking motility

Hitchhiking motility was assayed according to a method described elsewhere (Samad et al., 2017) but with modifications. *Staphylococcus aureus* and *Pseudomonas aeruginosa* strains were cultured to the mid-log phase and then mixed at a ratio of 1:100 in TSB. Bacterial mixture (200 μl) that contained 10^6^ colony forming unit (CFU) of *S. aureus* and 10^8^ CFU of *P. aeruginosa* was transferred into the wells in a Calgary Biofilm Device (CBD; Innovotech, Edmonton, Alberta, Canada). The plate was placed at room temperature for 1 h. Subsequently, the pegs on a CBD lid was immersed in the wells for 30 s to allow attachment of the bacteria. The pegs on the lid were then washed by immersing the pegs in 200 μl PBS in each well of a 96-well microtiter plate with gentle shaking using a Thermomixer (Eppendorf, Hamburg, Germany). After washing, the lid was transferred and immersed in a new 96-well plate containing 200 μl PBS and 0.1 mm-diameter glass beads (Biospec Products, Bartlesville, Oklahoma, United States). The plate was vortexed gently using a Thermomixer (Eppendorf, Hamburg, Germany) to remove the bacteria from the pegs. The bacterial suspension was then serially diluted and plated on mannitol salt agar. The number of *S. aureus* SA113 on the pegs was enumerated by viable cell count.

### Scanning electron microscopy

The pegs on the lid of CBD were removed and fixed overnight in 2% glutaraldehyde. The bacteria were examined under a field emission scanning electron microscope (FE-SEM, Hitachi SU8220, Hitachi High-Tech, Tokyo, Japan) according to a method described elsewhere (Samad et al., 2017). Images were colored using Photoshop CS3 (Adobe Systems, San Jose, California, United States).

### Swarming assay

TSA plates prepared with 0.4% agarose (TSA-0.4; Seakem, Lonza Rockland, Maine, United States) were used for swarming assay. Bacterial culture (2 μl) that contained 1×10^4^ CFU of *S. aureus* and 1 × 10^6^ CFU of *P. aeruginosa* was spotted at the center of a TSA-0.4 plate. The plate was subsequently incubated at 37°C for 4 h to allow *P. aeruginosa* to swarm. The bacteria at the center and the edge of a swarm were picked with a toothpick, which was inserted vertically into the bottom of the plate. The bacteria adhered to the toothpick were dissolved in 1 ml PBS. After serial dilution, bacteria were plated on mannitol salt agar. The number of *S. aureus* in the bacterial suspension was enumerated by viable cell count.

### Flow cytometry analysis

To detect the binding of LPS from *P. aeruginosa* to *S. aureus*, FITC-LPS (Sigma-Aldrich, United States) and free LPS (Sigma-Aldrich, United States) were mixed with 10^5^ CFU of *S. aureus*. The fluorescence intensity was detected, and the number of *S. aureus* SA113 bound to FITC-LPS was enumerated with a Guava easyCyte flow cytometer (Merck Millipore). To assess the binding of WTA of *S. aureus* or free lipoteichoic acids (LTA; Sigma-Aldrich, United States) to *P. aeruginosa*, mid-log phase *S. aureus* and *P. aeruginosa* were stained with hexidium iodide (Thermo Fisher Scientific, Waltham, MA, United States) and SYTO 9 (Invitrogen; Thermo Fisher Scientific) according to the instruction provided by the vendors. Labeled *S. aureus* and *P. aeruginosa* were mixed at a ratio of 1:1 and 10^5^ CFU/ml of the bacteria was used for flow cytometry analysis. Five thousand fluorescent-stained cells were counted in a gate set in a hexidium iodide versus SYTO 9 dot plot.

### *Caenorhabditis elegans* model

*Caenorhabditis elegans* N2 was cultured and maintained according to a method described previously (Brenner, 1974). *Staphylococcus aureus* strains harboring pCtuf-ppmch and SYTO 9-labeled *P. aeruginosa* were used in this study. *Staphylococcus aureus* and *P. aeruginosa* were subcultured for 4 h. Then, bacterial pellets were collected, mixed, and suspended in 200 μl PBS. The bacterial suspension was inoculated on TSA plates, and the inoculum was allowed to dry at room temperature. *Caenorhabditis elegans* at the L4 stage were placed onto bacteria-containing TSA plates and incubated at 20°C for 30 min. The worms were then transferred and mounted onto 2% agarose pads on a slide covered with 1% levamisole (Sigma-Aldrich, United States) to paralyze the worms. Bacteria in the digestive tracts of *C. elegans* were observed and imaged under a confocal laser-scanning microscope at 0 and 60 min after feeding, worms were imaged under a Olympus FV10i microscope. Meanwhile, the images were captured at 30 s interval for 5 min and converted into a time-lapse video. The distance of bacterial migration was measured and analyzed using FV10-ASW 4.2 software (Olympus).

### Mouse model

Six- to eight-week-old BALB/c mice were used in this study. The animal experiments were approved by the Chang Gung University Animal Care Committee (approval no. CGU108-191) and performed in accordance with the approved animal care guidelines and protocols. To improve the imaging quality, the backs of the mice were shaved. Mice were anesthetized by isoflurane inhalation and 50 μl of bacterial suspension, which contained 1 × 10^7^ CFU of luminescent *S. aureus* CGL1190 and 5 × 10^7^ CFU of *P. aeruginosa* PAO1, was injected subcutaneously into the dorsal region of the animals. The mice were then imaged with an IVIS instrument at 0 and 6 h post-injection. After observation, the mice were sacrificed, and the skin tissues at the injection site and the areas 2 cm away from the injection site were collected using a single hole puncher. The skin tissues were homogenized in 1 ml sterile PBS followed by centrifugation at 500 ×g for 5 min. The supernatant was serially diluted and plated on mannitol salt agar plates. The number of luminescent *S. aureus* was determined by viable cell count.

### Statistical analysis

The between-group differences were determined using two-tailed Student’s *t* test and one-way analysis of variance (ANOVA) test. According to the Shapiro–Wilk’s test, the results of *C. elegans* studies did not exhibit a normal distribution; the Kruskal-Wallis test was further used for statistical analysis of the *C. elegans* studies. All statistical analysis were performed using GraphPad Prism software version 8.0 (GraphPad Software, San Diego, California, United States).

## Results

### *Staphylococcus aureus* movement is facilitated by flagellated *Pseudomonas aeruginosa*

**Figure 1:**
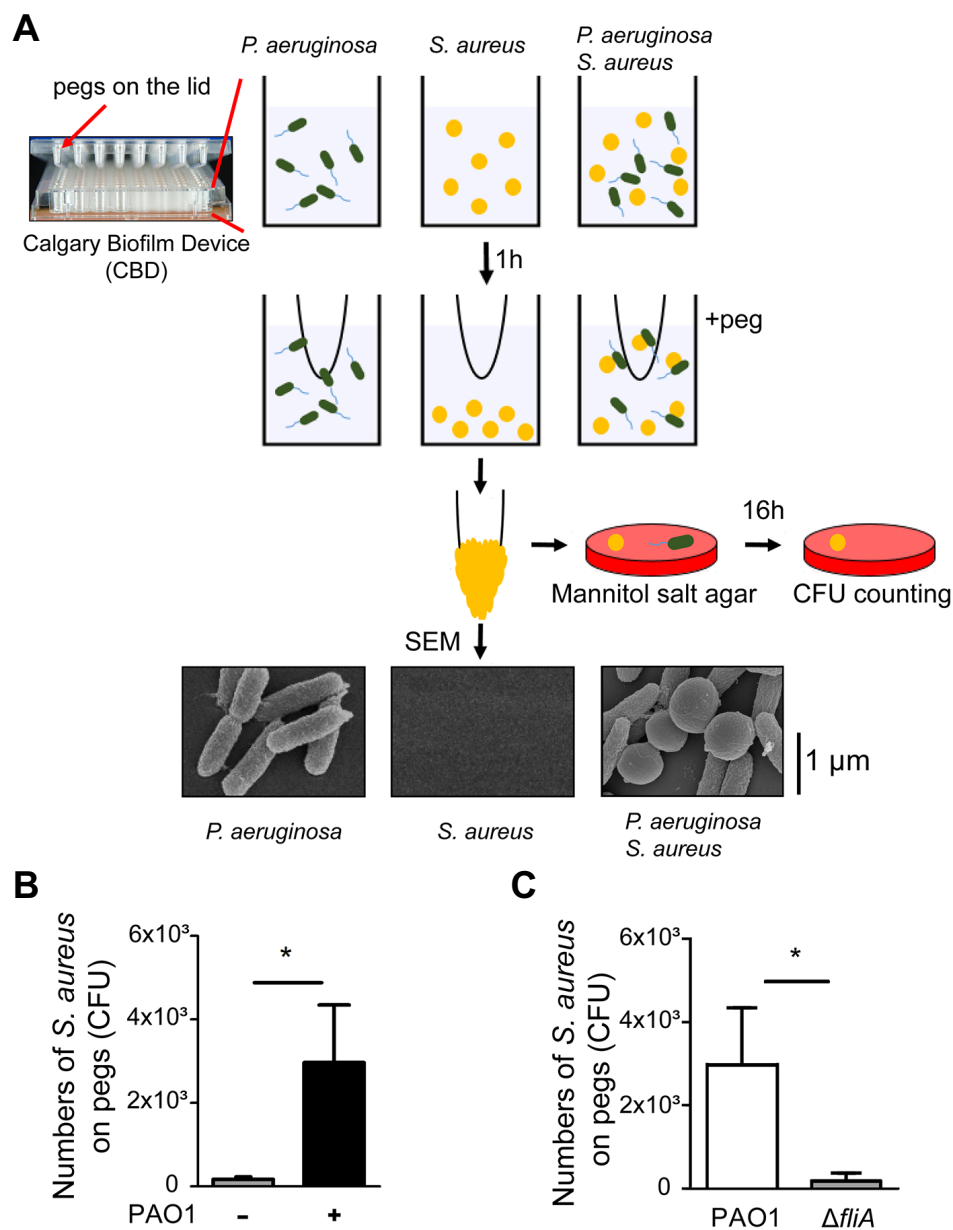
Promotion of *Staphylococcus aureus* movement by motile *Pseudomonas aeruginosa*. **(A)** Assay of *S. aureus* SA113 movement prompted by swimming *P. aeruginosa* PAO1. Bacteria were cultured in wells of CBD plates. After incubation for 1 h, the pegs on the lid were immersed into the culture for 30 s. *Staphylococcus aureus* adhered on the pegs was enumerated by viable cell count. Bacteria on pegs were also examined by SEM. **(B,C)** Wells were seeded with *S. aureus* SA113 only or mixed with *P. aeruginosa* PAO1 or its Δ*fliA* mutant strain. The number of *S. aureus* SA113 adhered on pegs was determined. Data are presented as the mean of the results from more than three independent experiments and were analyzed statistically using Student’s *t* test. Error bars indicate standard deviations. Significant differences (*p* < 0.05) are denoted with *.

An earlier study demonstrated that *S. aureus* achieves movement in a liquid environment by interacting with a swimming bacterium (Samad et al., 2017). A hitchhiking motility assay (Samad et al., 2017) was modified and used in this study to measure the ability of *P. aeruginosa* to carry *S. aureus* during swimming. We added a mixture of *S. aureu*s and *P. aeruginosa* into the wells of a CBD. After the bacterial mixture was stratified into two different layers with motile bacteria on the top and nonmotile bacteria at the bottom in the wells, we collected the bacteria on the top using the pegs on the lid of the CBD for CFU enumeration (Figure 1A). The results showed that when *S. aureus* SA113 was incubated in wells alone, the average CFU of *S. aureus* SA113 that adhered to a peg was 173. The CFU of bacteria on the pegs increased 17-fold to 2,966 CFU when *S. aureus* was incubated with *P. aeruginosa* PAO1 (Figure 1B). We also used a Δ*fliA* mutant of *P. aeruginosa* PAO1, which lacks flagellum. Compared with flagellated *P. aeruginosa* PAO1, the mutant carried 100 times less *S. aureus* SA113 to the pegs (Figure 1C), indicating that adherence of *S. aureus* to the pegs depends on the motility of *P. aeruginosa*. These results were consistent with an earlier finding that *S. aureus* is carried by *P. aeruginosa* to migrate (Samad et al., 2017). The attachment of *P. aeruginosa* PAO1 and *S. aureu*s SA113 to the pegs was also verified by SEM (Figure 1A).

### WTA of *Staphylococcus aureus* is required for the interaction with *Pseudomonas aeruginosa*

**Figure 2:**
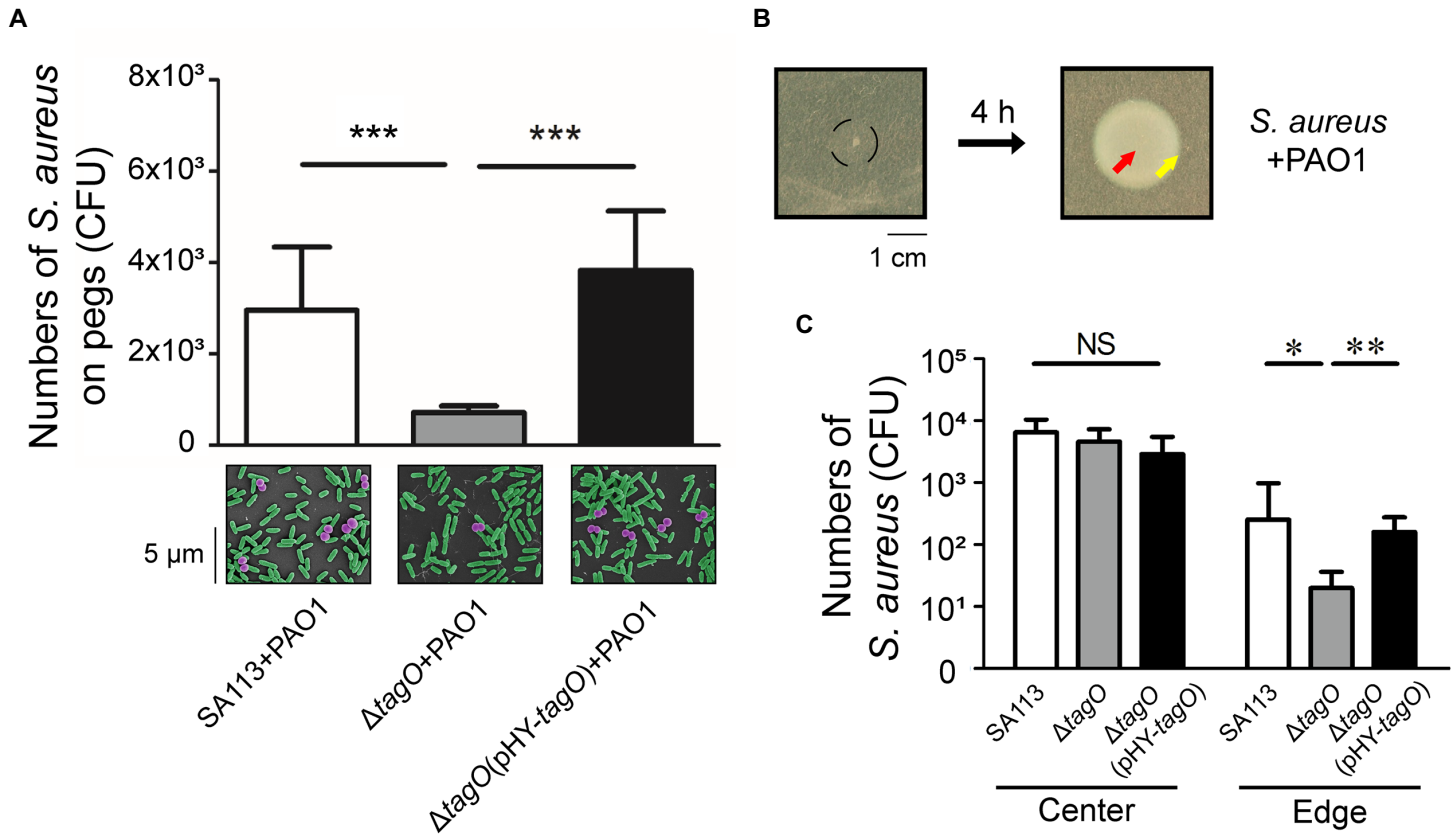
Influence of WTA in the hitchhiking motility of *Staphylococcus aureus*. **(A)** *Staphylococcus aureus* SA113, Δ*tagO* mutant of *S. aureus* SA113, and the mutant transformed with pHY-tagO were mixed with *Pseudomonas aeruginosa* PAO1 and added into wells of CBD plates. The number of *S. aureus* adhered on pegs was determined. The lower panel shows SEM images of *S. aureus* (purple) associated with *P. aeruginosa* (green) on pegs. The images shown are representative of three independent experiments. **(B)** *Staphylococcus aureus* was mixed with *P. aeruginosa* and inoculated on TSA-0.4 plates. The black circle indicates the area of inoculation (left panel). The plates were incubated for 4 h to allow bacteria to swarm. **(C)** *Staphylococcus aureus* located at the center (red arrow) and the edge (yellow arrow) of the swarm were collected using a toothpick. Bacteria on the toothpick were suspended in PBS and plated on mannitol salt agar for viable cell counts. Data are presented as the mean of the results from more than three independent experiments. Data were analyzed statistically using One-way ANOVA. Error bars indicate the standard deviations. Significant differences are denoted as follows: * indicates *p* < 0.05, ** indicates *p* < 0.01, and *** indicates *p* < 0.001. NS, no significance.

The interaction between *S. aureus* and *P. aeruginosa* suggested that surface components of *S. aureus* were involved in the hitchhiking motility. Our study found that the number of *S. aureus* that adhered to a peg was reduced after deleting *tagO* from strain SA113, which is required for the synthesis of wall teichoic acids (WTA; Weidenmaier et al., 2004). The results showed that when cocultured with *P. aeruginosa* PAO1, the average CFU of *S. aureus* SA113 that adhered to a peg was 3.1 × 10^3^ (Figure 2A). However, after *tagO* was deleted, the average CFU of bacteria adhered to a peg reduced 4.3-fold to 7 × 10^2^ (Figure 2A). The number increased to 3.8 × 10^3^ CFU per peg when the mutant strain was transformed with pHY-tagO, a plasmid expressing TagO (Figure 2A), showing the importance of WTA in the hitchhiking motility. Similar results were also observed by SEM (Figure 2A, lower panel).

The study further examined whether *P. aeruginosa* carried *S. aureus* during swarming. It is known that *S. aureus* spreads on soft agar surfaces, a movement resembles swarming (Kaito and Sekimizu, 2007). As *S. aureus* SA113 does not spread due to its *agr*-quorum sensing system is defective (Herbert et al., 2010; Tsompanidou et al., 2011), the movement of *S. aureus* SA113 during *P. aeruginosa* swarming could not be attributed to the spreading of *S. aureus*. Two microliter bacterial culture that contained 1 × 10^4^ CFU of *S. aureus* and 1 × 10^6^ CFU of *P. aeruginosa* was spotted at the center of a TSA-0.4 plate. Four hours after inoculation on agar medium with a mixture containing *S. aureus* SA113 and *P. aeruginosa* PAO1, the bacteria at the center and the edge were picked with a toothpick (Figure 2B). The results showed that the average CFU of *S. aureus* SA113, Δ*tagO* mutant of SA113, and the Δ*tagO* mutant of SA113 transformed with pHY-tagO at the center were similar (Figure 2C). However, at the edge of the swarm, average CFU of *S. aureus* SA113, the Δ*tagO* mutant, and the mutant harboring pHY-tagO were 251, 20 and 158 CFU, respectively (Figure 2C). The results indicated that *S. aureus* SA113 does not migrate to the edge of a swarm efficiently after *tagO* is deleted. According to the results from Figures 1, 2A, *P. aeruginosa* interacted with WTA of *S. aureus* and carried *S. aureus* to move around in the liquid environment, which facilitated *S. aureus* up to top layer and adhered to pegs. The phenomenon of WTA dependent carriage between *P. aeruginosa* and *S. aureus* is also observed on surface. Meanwhile, the whole swarm colony was harvested for CFU counting. The CFU of *S. aureus* increased from 1 × 10^4^ to 1 × 10^5^, indicating that during co-incubation with *P. aeruginosa* for 4 h, *S. aureus* still multiplied.

### LPS of *Pseudomonas aeruginosa* is involved in the interaction with WTA of *Staphylococcus aureus*

**Figure 3:**
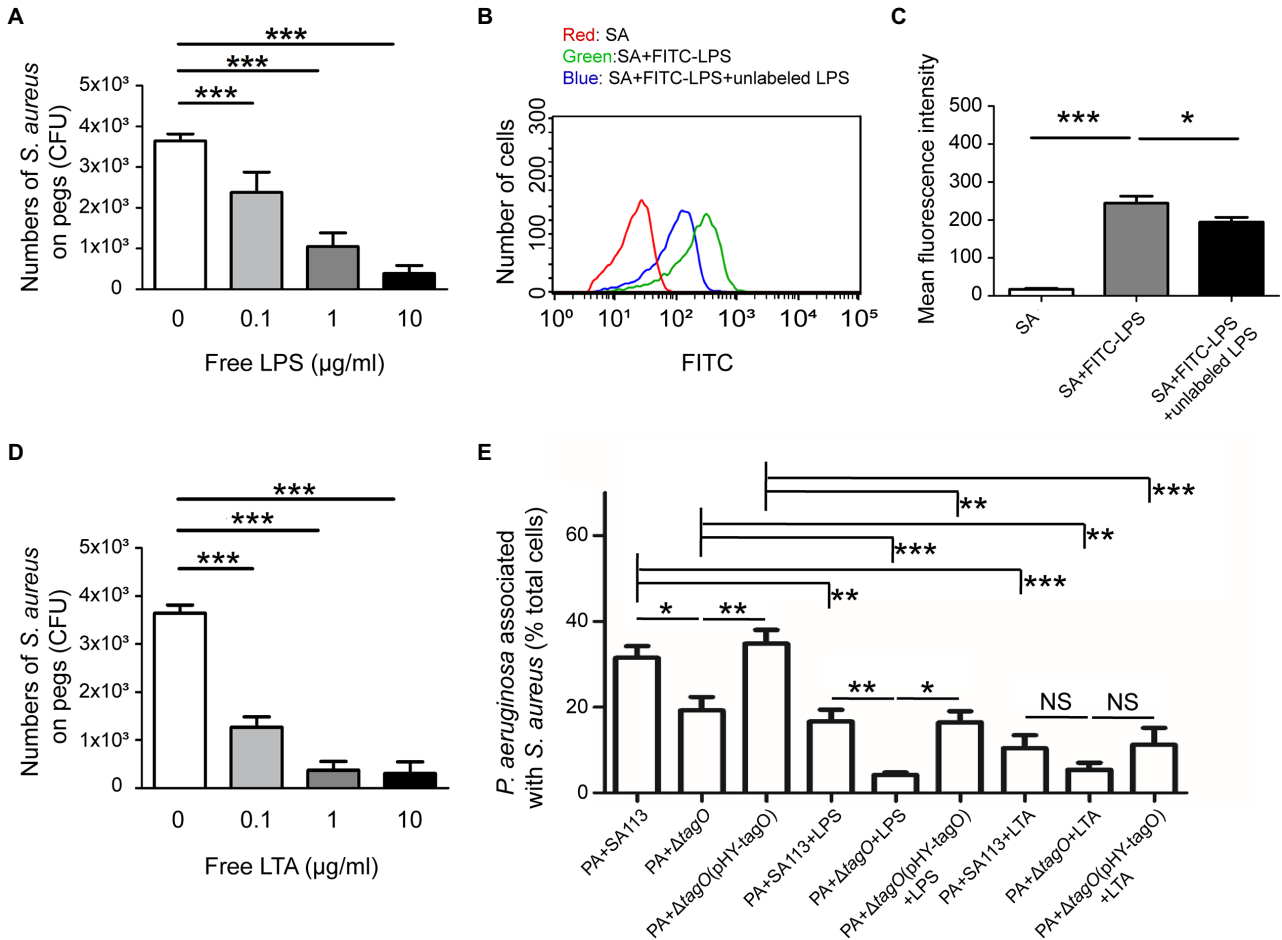
Interaction between WTA of *Staphylococcus aureus* and LPS of *Pseudomonas aeruginosa. Staphylococcus aureus* SA113 was mixed with various concentrations of free LPS **(A)** or LTA **(D)** for 10 min, and then mixed with *P. aeruginosa* PAO1. The mixtures were seeded into the wells of CBD plates. The number of *S. aureus* on pegs was determined. **(B)** *Staphylococcus aureus* SA113 (SA) was incubated alone (red) or with FITC-LPS in the absence (green) or presence (blue) of unlabeled LPS. The binding of FITC-LPS to *S. aureus* SA113 was analyzed by flow cytometry. The representative histogram of flow cytometry was shown. **(C)** Mean of fluorescence intensity of FITC-LPS bound *S. aureus* was measured and analyzed using Guavasoft 3.3. **(E)** *Pseudomonas aeruginosa* and *S. aureus* strains were labeled with SYTO 9 and hexidium iodide, respectively. *Pseudomonas aeruginosa* was incubated with *S. aureus* in the absence or presence of free LPS or LTA. After incubation, the cells were analyzed by flow cytometry. The data shown are the average percentage of *P. aeruginosa* associated with *S. aureus* in total analyzed *P. aeruginosa*. Bar presents as the mean of the results from more than three independent experiments and analyzed statistically using One-way ANOVA. Error bars indicate the standard deviations. Significant differences are denoted as follows: * indicates *p* < 0.05, ** indicates *p* < 0.01, and *** indicates *p* < 0.001.

An earlier study showed that *Bifidobacterium* interacts with the LPS of *E. coli* to neutralize its toxicity (Park et al., 2007), implying that LPS is a potential candidate involved in associations with Gram-positive bacteria. To investigate the components of *P. aeruginosa* that interacted with WTA of *S. aureus*, free LPS from *P. aeruginosa* was used in the hitchhiking motility assay according to the method described in Figure 1A. The results showed that when free LPS was mixed with *S. aureus* before coincubation with *P. aeruginosa,* the number of *S. aureus* that adhered to a peg decreased in a dose-dependent manner (Figure 3A). Average CFU of *S. aureus* that adhered to the pegs was 3.6 × 10^3^. The CFU decreased to 2.4 × 10^3^, 1.1 × 10^3^, and 0.4 × 10^3^ when the bacteria were cultured in TSB containing 0.1, 1, and 10 μg/ml free LPS, respectively (Figure 3A). The results indicated that adding LPS competes for the binding of *S. aureus* to *P. aeruginosa*. Flow cytometry analysis also verified the binding of FITC-LPS to *S. aureus* (Figure 3B). The results demonstrated that the mean fluorescence intensity (MFI) of *S. aureus* SA113 alone was 19. The MFI increased to 253 when *S. aureus* SA113 was incubated with FITC-LPS. When unlabeled LPS was added, the MFI of FITC-LPS bound *S. aureus* decreased to 186 (Figure 3C). To assess whether WTA of *S. aureus* was required for the binding to LPS of *P. aeruginosa*, free LPS, hexidium iodide-labeled *S. aureus* SA113, Δ*tagO* mutant of SA113, and the mutant transformed with pHY-tagO and SYTO 9-labeled *P. aeruginosa* were used in flow cytometry analysis. The results demonstrated that when *S. aureus* SA113 was mixed with *P. aeruginosa* at a ratio of 1:1, the average percentage of *P. aeruginosa* associated with *S. aureus* SA113 was 32 (Figure 3E). When *tagO* was deleted from strain SA113, the average percentage of *P. aeruginosa* associated with the mutant reduced to 21.8 (Figure 3E); but increased to 34.7 when the mutant strain was transformed with pHY-tagO (Figure 3E). We also added free LPS to the mixture to determine whether LPS competes for the binding of *S. aureus* to *P. aeruginosa*. The results showed that compared with the strains untreated with LPS, the addition of free LPS decreased the binding percentage of *P. aeruginosa* to *S. aureus* SA113, the SA113 Δ*tagO* mutant and the mutant transformed with pHY*-*tagO from 32, 21.8, and 34.7 to 16, 6.3, and 18, respectively (Figure 3E). Furthermore, we examined whether lipoteichoic acids (LTA), which is a teichoic acid that anchored to the membrane lipid, is involved in the interaction between *S. aureus* and *P. aeruginosa*. When free LTA from *S. aureus* was used in the hitchhiking motility assay, the similar results were obtained as that of LPS (Figure 3D). The results showed that adding free LTA competes for the binding of *S. aureus* to *P. aeruginosa* in a dose-dependent manner (Figure 3D). Flow cytometry analysis also confirmed the percentage reductions after adding LTA. The results demonstrated that adding free LTA decreased the average percentage of the binding of *P. aeruginosa* to *S. aureus* SA113, the SA113 Δ*tagO* mutant, and the mutant transformed with pHY*-*tagO from 32, 21.8, and 34.7 to 10.4, 7.9, and 10.8 (Figure 3E), verifying that *P. aeruginosa* interacts with *S. aureus via* the interaction of LPS with WTA or LTA (Figure 3E). However, in comparison with *S. aureus* SA113, addition of free LTA to the Δ*tagO* mutant of SA113 or the mutant transformed with pHY*-*tagO, the differences of the binding of *P. aeruginosa* to *S. aureus* is not statistically significant between the groups (Figure 3E). The results suggested that WTA and LTA target different components on the *P. aeruginosa* surface for interaction.

### Hitchhiking motility of *Staphylococcus aureus* SA113 prompted by *Pseudomonas aeruginosa* PAO1 in *Caenorhabditis elegans*

After demonstrating that *P. aeruginosa* PAO1 promotes the movement of *S. aureus* SA113 on surface, this study further investigated whether *P. aeruginosa* facilitates the movement of *S. aureus* in a *C. elegans* model. In this study, *S. aureus* SA113, the SA113 Δ*tagO* mutant and the mutant with pHY-tagO were transformed with pCtuf-ppmch to express mCherry (Mauthe et al., 2012). Meanwhile, *P. aeruginosa* PAO1 was stained with SYTO 9 to exhibit green fluorescence.

**Figure 4:**
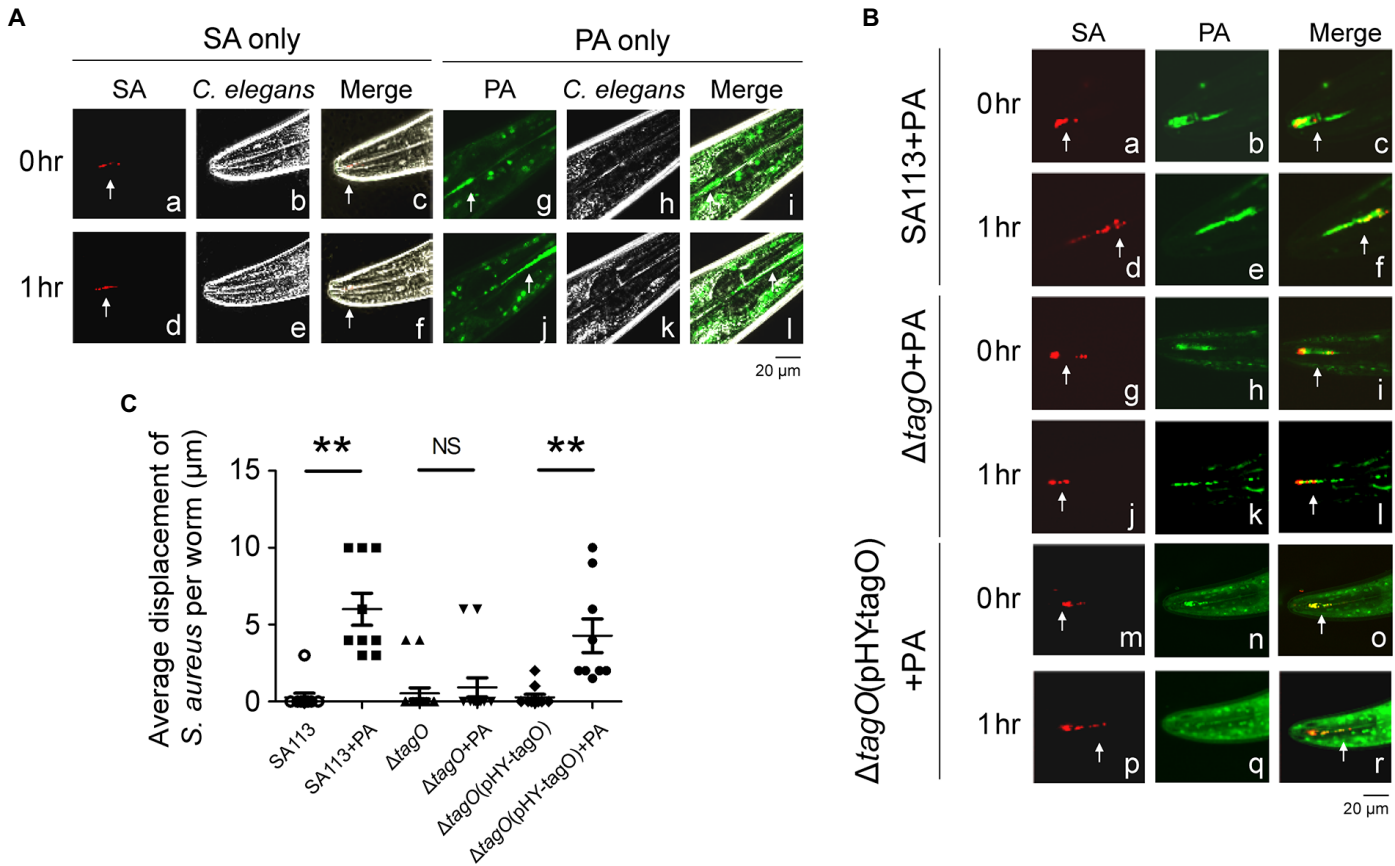
*Pseudomonas aeruginosa* promoted the movement of *Staphylococcus aureus* in *Caenorhabditis elegans*. **(A)** *Caenorhabditis elegans* were placed on TSA plates that had been inoculated with *S. aureus* SA113(pCtuf-ppmch; SA) or CYTO 9-labeled *P. aeruginosa* PAO1 (PA). **(B)** CYTO 9-labeled *P. aeruginosa* PAO1 (PA) was mixed with *S. aureus* SA113(pCtuf-ppmch) (a–f), Δ*tagO*(pCtuf-ppmch) (g–l) and Δ*tagO*(pHY-tagO, pCtuf-ppmch) (m–r) and inoculated on TSA plates. The worms were placed on the bacteria-containing agar, and fluorescence (white arrow) in the digestive tracts of *C. elegans* was imaged under a confocal laser-scanning microscope. Another image was obtained at the same location of the worm 1 h later. Autofluorescence (green) was observed in the *C. elegans* background. Phase-contrast microscopy images showed the shape of the worm. Merged images showed that bacteria were located inside *C. elegans*. The images shown are representative of each group (*n* ≥ 7 per group). **(C)** The distance of bacterial migration was measured using FV10-ASW 4.2 software. Data are presented as the mean of the results from each group and were analyzed statistically using Kruskal–Wallis test. Error bars indicate standard deviations. Significant differences (*p* < 0.01) are denoted as **. NS, no significance.

*Caenorhabditis elegans* was fed with bacteria and the movement of the bacteria in the digestive track was followed and imaged. The images revealed that when the worms were fed with *S. aureus* SA113, little movement of the bacteria was observed; the bacteria at the same location during an hour; the bacteria moved only 0.3 μm within an hour (Figures 4Aa–f, C). When the worms were fed a mixture of *S. aureus* SA113 and *P. aeruginosa* PAO1, *S. aureus* SA113 moved along with *P. aeruginosa* PAO1 with a distance of 6.0 μm (Figures 4Ba–f, C). When the worms were fed with a bacterial mixture containing the Δ*tagO* mutant of SA113 and *P. aeruginosa* PAO1, little movement of the mutant was observed; the mutant moved only 0.9 μm (Figures 4Bg–l, C). However, the mutant was found to co-migrate with *P. aeruginosa* PAO1 and reached a distance of 4.3 μm at 1 h after feeding if the mutant was transformed with pHY-tagO to complement the mutation (Figures 4Bm–r, C). Time-lapse video showed that *S. aureus* (red) moved together with *P. aeruginosa* (green; Supplementary Videos S1, S2). However, due to prolonged exposure to laser damaged the digestive tract and was lethal to the worm, this study was unable to collect the results beyond the 1 h period.

### Exhibiting hitchhiking motility of *Staphylococcus aureus* SA113 in a mouse model

**Figure 5:**
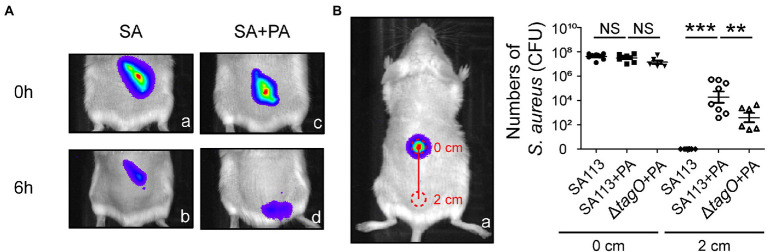
Movement of *Staphylococcus aureus* mediated by *Pseudomonas aeruginosa* in mice. **(A)** Mice were injected subcutaneously with bioluminescent *S. aureus* CGL1190 (SA) alone **(a,b)** or SA mixed with *P. aeruginosa* PAO1 (SA + PA) **(c,d)**. Bioluminescence of SA in mice was observed and imaged at 0 and 6 h post-inoculation using an IVIS instrument. The images shown are representative of each group (*n* = 8 per group). **(B)** After observation, the mice were sacrificed, and skin tissue at areas of 0 and 2 cm (red circles) was removed. The number of *S. aureus* recovered from the skin tissues was determined. Data are presented as the mean of the results from each group and were analyzed statistically using One-way ANOVA. Error bars indicate the standard deviations. Significant differences are denoted as follows: ** indicates *p* < 0.01, and *** indicates *p* < 0.001. NS, no significance.

*Staphylococcus aureus* and *P. aeruginosa* are commonly associated with wound infection (DeLeon et al., 2014). To clarify whether *P. aeruginosa* promotes spreading of *S. aureus* in skin tissues, we used IVIS and observed the movement of *S. aureus* CGL1190, a strain exhibiting bioluminescence, in mice. The results showed that bioluminescence signals were present in the injection site, indicating that the bioluminescent *S. aureus* was useful in a mouse skin model (Figures 5Aa,c). Compared to inoculations with *S. aureus* alone, *S. aureus* spread farther when co-inoculated with *P. aeruginosa* (Figures 5Ab,d). To quantify the number of *S. aureus* moved away from the original injection site, the skin tissues at the inoculated areas and the areas 2 cm away were removed from the mice to enumerate the bacteria in the regions. The results showed that at 6 h after inoculation with *S. aureus* SA113 alone, the tissue in the area 2 cm away from the inoculation site contained only a few *S. aureus*, the average CFU was 3 (Figure 5B). In contrast, when inoculated with the bacterial mixture containing *S. aureus* SA113 and *P. aeruginosa* PAO1, the average CFU of *S. aureus* in the skin tissue 2 cm away from the inoculation site was 1.9 × 10^4^ (Figure 5B). However, the average CFU of *S. aureus* was 1.5 × 10^3^ CFU when inoculated with a mixture containing the Δ*tagO* mutant and *P. aeruginosa* PAO1 (Figure 5B). The results indicated that *P. aeruginosa* promotes the movement of *S. aureus* in the mouse skin model. The results also showed that WTA is required for hitchhiking *P. aeruginosa* by *S. aureus* SA113 *in vivo*. Furthermore, we plated 50 μl blood collected from the mice at 6 h post-inoculation, on mannitol salt agar plates and did not detected any *S. aureus* and *P. aeruginosa* in the blood. Additionally, our IVIS study revealed that the bioluminescence was largely confined near the tail, rather than the whole body suggesting that bacteria movement does not involve the dispersion of the bacteria *via* the circulatory system. The results excluded the possibility that the bacterial dissemination was due to the entry of the bacteria into the bloodstream.

## Discussion

Motility is crucial for bacteria to travel in the environment. Due to the lack of pili and flagellum, *S. aureus* is nonmotile. Although *S. aureus* does not move actively, the bacterium uses a mechanism similar to that of *B. subtilis* swarming to extract water from agar medium and expresses biosurfactants, such as PSMs, to weaken the water surface tension, thereby facilitating colony spreading (Ke et al., 2015; Lin et al., 2016). Additionally, like *B. subtilis*, an *S. aureus* spreading colony often forms dendrites during spreading (Pollitt et al., 2015). An earlier study demonstrated that *S. aureus* associates with flagellated bacteria, such as *P. aeruginosa,* to facilitate hitchhiking motility (Samad et al., 2017). This study investigates how *S. aureus* hitchhikes *P. aeruginosa* for movement.

TagO is an enzyme required for the synthesis of WTA, which is a major surface component of Gram-positive bacteria (Brown et al., 2013). WTA of *S. aureus* is important to biofilm formation (Vergara-Irigaray et al., 2008) and colonizing the human skin (Burian et al., 2021). Moreover, a receptor for the binding of staphylococcal WTA has been identified on human nose epithelial cells (Baur et al., 2014). WTA also serves as key factors for recognition by bacteriophages (Eugster and Loessner, 2012). These studies indicated that WTA of *S. aureus* has a strong binding ability to different molecules. Indeed, our study found that *tagO* mutation decreased the hitchhiking motility of *S. aureus* prompted by *P. aeruginosa* (Figures 2, 3). In addition to WTA, we found that LTA, another important cell wall polymer found in Gram-positive bacteria, is also involved in interaction between *P. aeruginosa* and *S. aureu*s (Figures 3D, E). However, whether LTA binds to LPS of *P. aeruginosa* remaining unclear. Nevertheless, involvement of LTA in binding to *P. aeruginos*a may explain why WTA deficiency alone does not completely abolish the binding of *P. aeruginosa* to *S. aureus* (Figure 2). We also examined whether factors other than WTA and LTA in *S. aureus* are involved in the interaction with *P. aeruginosa*. We found that a mutant strain of SA113 that is defective in fibronectin-binding protein (FnBP) synthesis (unpublished) interacts with *P. aeruginosa* PAO1 at a level comparable to the parental strain, showing that FnBP is uninvolved in the interaction (data not shown).

Both WTA and LTA of *S. aureus* are polyanionic polymers, modification of the backbone of the polymer with D-alanine, mono- or oligosaccharides has been shown to change surface properties of *S. aureus*, which provides structures for binding or interacting with a variety of receptors (van Dalen et al., 2020). D-alanylation is known to increase the positive charge of the polymers and confers them the zwitterionic properties, which are important for interacting with their binding partners (Collins et al., 2002; Weidenmaier et al., 2010). In addition to D-alanylation, glycosylation alters physiological properties of the polymers and influences their binding with extracellular molecules (Winstel et al., 2015; Gerlach et al., 2018). According to the results from flow cytometry analysis (Figure 3E), although the binding ability of *S. aureus* SA113 to *P. aeruginosa* PAO1 reduces after *tagO* mutation, the binding is restored if the mutation is complemented. Furthermore, adding free LTA decreases the binding between *P. aeruginosa* PAO1 and the *tagO* mutant (Figure 3E). The level of decrease is comparable to the reduction observed between *P. aeruginosa* PAO1 and *S. aureus* SA113 after adding LTA (Figure 3E), suggesting that WTA and LTA are two independent factors affecting the binding of *S. aureus* to *P. aeruginosa*. Therefore, we propose that D-alanylation increases the positive charge of WTA thus reducing the repulsion between WTA and LPS, which is also a polyanionic polymer on surface of Gram-negative bacteria. When WTA is near LPS, the interaction between glycan modification of WTA and LPS occurs.

LPS is a structure that functions similarly to WTA (Huszczynski et al., 2019). In addition to being a virulence factor, LPS is an important ligand for bacterial adhesion (Walker et al., 2004; Huszczynski et al., 2019). An earlier study showed that *Bifidobacterium* binds to LPS in *E. coli* (Park et al., 2007). In the present study, LPS supplementation competes for the binding of *S. aureus* to *P. aeruginosa*. Flow cytometry analysis also showed that WTA of *S. aureus* is required for binding to LPS. The results suggested that the binding of WTA of *S. aureus* to LPS of *P. aeruginosa* is the underlying mechanism for the hitchhiking motility of *S. aureus*. Although WTA and LPS are two major components involved in the interaction between *S. aureus* and *P. aeruginosa*, this study found that free LPS cannot completely abolish the binding of *S. aureus* to *P. aeruginosa* (Figure 3E), indicating that factors other than LPS on *P. aeruginosa* may be involved in interacting with *S. aureus*.

This study found that in addition to *P. aeruginosa*, other motile bacteria, such as *E. coli*, and even motile Gram-positive bacteria, such as *B. subtilis* and *Listeria monocytogenes,* also promote the hitchhiking motility of *S. aureus*, although less efficiently than *P. aeruginosa* (Supplementary Figure S1). Teichoic acids or related polymers are known to bind to cell wall proteins, S-layers, or mycolic acids in other Gram-positive bacteria (Jonquieres et al., 1999; Zoll et al., 2012). It is plausible that the interactions between Gram-positive bacteria involve these factors. However, adding free LTA into the cultures containing *S. aureus* and *L. monocytogenes* does not impair the hitchhiking motility of *S. aureus* (data not shown), indicating that LTA is unlikely involved in the interaction between *S. aureus* and *L. monocytogenes*. Furthermore, this study finds that these motile bacteria although support the hitchhiking motility of *S. aureus* SA113, do not fully assist the movement of the Δ*tagO* mutant of *S. aureus* (Supplementary Figure S1), indicating that WTA of *S. aureus* is a crucial factor associated with either Gram-negative or Gram-positive bacteria. The results suggested that *S. aureus* using WTA to hitchhike on motile bacteria is a general strategy for its dispersal.

Coinfection with *P. aeruginosa* and *S. aureus* is common in chronic lung infection and wound infections (Yung et al., 2021). A recent study showed that *P. aeruginosa* is capable of sensing *S. aureus* from a distance and initiates single-cell movement to invade the colony and eventually inhibits the growth of *S. aureus*. The study demonstrated that secreted factors controlled by the Agr quorum sensing system of *S. aureus* trigger the exploratory motility of *P. aeruginosa* toward *S. aureus* (Limoli et al., 2019). Due to *S. aureus* SA113 lacks the Agr quorum sensing system, the strain is unlikely to influence the movement of *P. aeruginosa* in our study. Furthermore, another single-cell study showed different results, which indicated that *S. aureus* are highly competitive against *P. aeruginosa* on surfaces (Niggli et al., 2021). The study demonstrated that when *P. aeruginosa* contacts with *S. aureus*, the contact enhances the growth of *P. aeruginosa* and activates the quorum sensing system of *P. aeruginosa* to inhibit *S. aureus* (Niggli et al., 2021). However, in our swarming and mouse studies, we did not observe the inhibition of *S. aureus* SA113 growth by *P. aeruginosa* PAO1 (Figures 2, 5), suggesting that interspecies interactions under co-culture conditions may not reflect the phenomena observed in the single-cell studies. However, several co-culture studies also showed that *P. aeruginosa* produces anti-staphylococcal compounds such as siderophores, rhamnolipids, and phenazines to inhibit the growth of *S. aureus* (Haba et al., 2003; Mashburn et al., 2005; Bharali et al., 2013; Borrero et al., 2014; Filkins et al., 2015); the mucoid *P. aeruginosa* produces exogenous alginate, which downregulates the expression of antistaphylococcal compounds, leading to protection of *S. aureus* from killing by *P. aeruginosa* (Limoli et al., 2017; Price et al., 2020). A transcriptomic study revealed that coexistence with *P. aeruginosa* alters the *S. aureus* transcriptome and its virulence (Briaud et al., 2019). These studies indicate that the interspecies interactions and coexisting mechanisms of *P. aeruginosa* and *S. aureus* are very complicate. The microorganisms change their moving behavior, gene expression and even lifestyle to achieve a competitive niche. Base on used bacterial strains, different culture conditions and study models, the conclusions derived from the studies may not be consistent. Despite the inconsistency, the studies, nevertheless, provide useful information on how bacteria use the interaction to survive in an ecosystem.

In this study, we showed that *S. aureus* hitchhikes flagellated *P. aeruginosa* to spread by interaction between WTA of *S. aureus* and LPS of *P. aeruginosa.* The hitchhiking motility promotes the migration of *S. aureus* in a *C. elegans* model as well as in mice, showing that the motility occurs not only in culture but also in animals. The overall results demonstrated how a mobile bacterium carries another motionless pathogen to spread in the environment and hosts in which provide useful information for developing strategy to treatment of chronic polymicrobial infections.

## Data availability statement

The original contributions presented in the study are included in the article/Supplementary material, further inquiries can be directed to the corresponding author.

## Ethics statement

The animal study was reviewed and approved by Chang Gung University Animal Care Committee.

## Author contributions

M-HL and C-CL conceived and designed the study, performed the experiments, analyzed the data, and wrote the manuscript. All authors contributed to the article and approved the submitted version.

## Funding

This work was supported by grants from Chang Gung Memorial Hospital (CMRPD1F0061-3 and CMRPD1J0271-3) and the Ministry of Science and Technology (MOST), Taiwan (MOST 108-2320-B-182-028).

## Conflict of interest

The authors declare that the research was conducted in the absence of any commercial or financial relationships that could be construed as a potential conflict of interest.

## Publisher’s note

All claims expressed in this article are solely those of the authors and do not necessarily represent those of their affiliated organizations, or those of the publisher, the editors and the reviewers. Any product that may be evaluated in this article, or claim that may be made by its manufacturer, is not guaranteed or endorsed by the publisher.
