# Supplementary material for: Hitchhiking motility of Staphylococcus aureus involves the interaction between its wall teichoic acids and lipopolysaccharide of Pseudomonas aeruginosa
Source: Front Microbiol. 2023 Jan 5;13:1068251. doi: 10.3389/fmicb.2022.1068251 (PMC9849799; doi:10.3389/fmicb.2022.1068251)
Supplement: Supplementary file 3 [file Data_Sheet_1.PDF]

Supplementary Material

Supplementary Figure S1

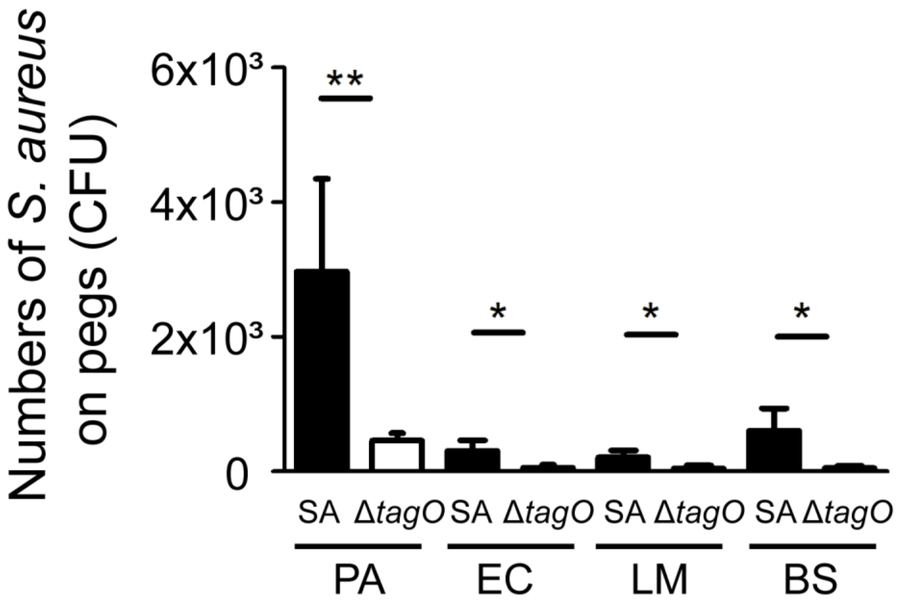

Supplementary Figure S1. Motile bacteria promote alternative spreading of *S. aureus*. *S. aureus* SA113 (SA) and *S. aureus* SA113Δ*tagO* (Δ*tagO*) were mixed with *P. aeruginosa* (PA), *E. coli* (EC), *Listeria monocytogenes* (LM) or *Bacillus subtilis* (BS). Bacterial mixtures were added to wells in a CBD plate. The number of *S. aureus* on pegs was determined as described in Fig. 1. Data are presented as the mean of the results from more than three independent experiments. Error bars indicate the standard deviations. Data were analyzed statistically using Student's *t* test. Significant differences are denoted as follows: * indicates $p<0.05$ and ** indicates $p<0.01$.

Supplementary Video S1 and S2 *In vivo* mobility of *S. aureus* facilitated by hitchhiking on *P. aeruginosa*. Time-lapse images were acquired under a confocal laser-scanning microscope at 30 s intervals for 5 min. The video shows that *S. aureus* SA113 (red) was associated with *P. aeruginosa* PAO1 (green) and moved together.
